# Frontline Account: Physician Partners: An Antidote to the Electronic Health Record

**DOI:** 10.1007/s11606-016-3727-x

**Published:** 2016-05-20

**Authors:** David B. Reuben, Niki Miller, Eve Glazier, Brandon K. Koretz

**Affiliations:** Division of Geriatrics, David Geffen School of Medicine at UCLA, 10945 Le Conte Ave., Suite 2339, Los Angeles, CA 90095-1687 USA; Division of General Internal Medicine and Health Services Research, David Geffen School of Medicine at UCLA, Los Angeles, CA USA

**Keywords:** primary care redesign, medical scribes, physician efficiency

## BACKGROUND

The modern medical record was originally developed in the 1920s as a way for physicians to briefly document patients’ medical conditions and plans for treating them. It was a means of jogging the memory so that solo practitioners could care for thousands of patients and quickly get up to speed on the major issues affecting each. Records were handwritten, lacked a consistent method of organization, and were often illegible to others. As medicine became more complicated and physician groups were formed, it became increasingly important for others to be able to view records, and the structure of the medical note became more standardized. Over time, the medical record began to be used for other purposes, such as for insurers who required documentation to justify reimbursement rates. With the advent of the electronic health record (EHR), the reasons for use have expanded dramatically, including documenting and improving quality of care, scheduling, billing, research, rapid communication within the health system and between patients and physicians, and tracking when and how long physicians are working. In short, the EHR has taken control of physicians’ professional lives.

In response, many physicians have become stressed and feel overburdened in practice.^1^ To cope with the additional work of documentation, physicians have changed how they interact with the patient, sitting at the keyboard, frequently with eyes on the screen rather than on the patient. They talk less and multitask more, searching for needed information in real time, further eroding the doctor–patient relationship. Some physicians have retired early rather than practice in the new EHR world. Others have off-loaded documentation and other administrative tasks to less highly trained personnel, such as scribes.^2^ In 2012, we first heard of these approaches and thought that they might be able to help relieve physician stress locally resulting from a push to see more patients and impending deployment of a new EHR. Accordingly, we built upon existing scribe programs to create the UCLA Physicians Partner program, with the intent of making the physician’s work easier, improving the quality of time spent with patients, and increasing efficiency.

## THE PHYSICIAN PARTNER PROGRAM

**Figure 1:**
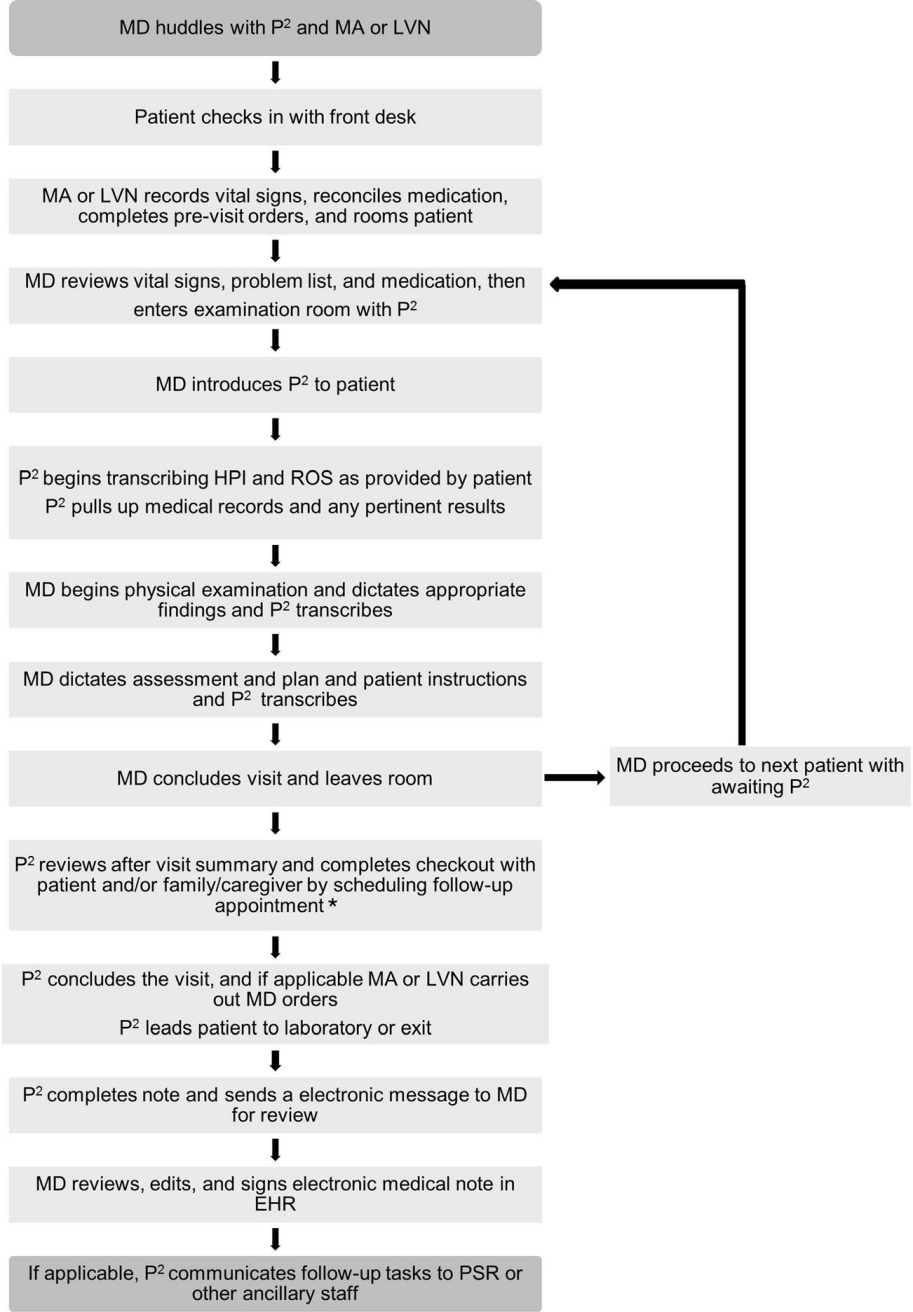
Choreography and roles of physician partners. *EHR* electronic health record, *HPI* history of present illness, *LVN* licensed vocational nurse, *MA* medical assistant, *MD* physician, *P* ^*2*^ Physician Partner, *PSR* patient services representative, *ROS* review of systems. ^a^ For general internal medicine visits, the P^2^ did not perform the check-out function in the exam room, and referred the patient to the front desk to perform these tasks.

The roles of Physician Partners include navigating the EHR, documenting patient encounters, and expediting patient care immediately prior to, during, and after the office visit. We initially recruited Physician Partners from existing personnel, two with bachelor’s degrees and the third a licensed vocational nurse. The three Physician Partners were paired with two physicians practicing concurrently in a 4-h clinic session, allowing for more continuous workflow (Fig. 1).

Prior to the start of the clinic session, the Physician Partners, along with the medical assistant or licensed vocational nurse, huddle with the physicians to prepare and discuss the scheduled patients. After the huddle, the physician and one of the Physician Partners enter the exam room. During the exchange of greetings with the patient, the physician introduces the Physician Partner and explains that she will assist during the visit by writing the note and placing any orders, allowing the physician to focus on the patient’s concerns.

Stationed at the computer, the Physician Partner listens to the conversation between the physician and patient, noting pertinent positives and negatives in the history of present illness, and populates the past medical history, review of systems, physical exam findings, the assessment and plan, and patient instructions. If the physician begins discussing recent lab tests or imaging, the Physician Partner opens the results in the EHR for physician reference, while simultaneously documenting the discussion. If the physician indicates that the patient will need additional tests or imaging, the Physician Partner queues up the order into the EHR for the physician to sign later.

At the conclusion of the visit, the physician instructs the patient to stay in the room to complete the check-out process with the Physician Partner. After the physician leaves, the Physician Partner schedules a follow-up visit and provides the patient with an after-visit summary detailing the visit and instructions to follow. When check-out is complete and the patient has left, the Physician Partner finishes the documentation and sends the note to the physician electronically for review.

## EFFECTS OF THE PROGRAM

We tested this new position as a pilot in an academic health center in geriatrics (three physicians) and general internal medicine (two physicians) practices, in the context of a newly implemented EHR system to determine its effect on physician efficiency and patient satisfaction and to confirm findings from an earlier pilot of the program using a different EHR.^3^

From September 2013 to December 2013, we collected time-series data to measure physician efficiency by calculating physicians’ time spent in the examining room during encounters with and without Physician Partners. In addition, physicians were asked to log the amount of time spent on documentation and administrative duties prior to and after a 4-h clinic session and to complete a survey evaluating satisfaction in working with a Physician Partner. Patients were also surveyed about the program.

In the geriatrics practice, 93 visits that included Physician Partners were an average of 4 min shorter per patient compared to 90 visits without Physician Partners (*P* = 0.0004), for a total of 48 min saved per 4-h session. The total time saved with a Physician Partner, including physician time spent in preparation prior to and post sessions, was 88 min per 4-h session. In the general internal medicine practice, 90 visits that included Physician Partners were an average of 2 min shorter per patient compared to 71 visits without Physician Partners (*P* = 0.016). General internists saved 92 min by working with a Physician Partner before and after each 4-h session.

Thirty physicians were surveyed, including the pilot physicians and others who had experienced one or more sessions with a Physician Partner; 97 % indicated that they placed great value on working with the Physician Partners, and 70 % indicated that they would be willing to add two patients per session to their schedule to do so. Of the 125 patients surveyed, 93 % responded “no” when asked whether they felt uncomfortable having a Physician Partner in the room, and 86 % of patients felt that the Physician Partners helped their visits run smoothly.

## DISCUSSION

Although we demonstrated increased efficiency and improved satisfaction among physicians, there have been barriers to the adoption of the Physician Partner into the clinic workflow, including limited physician receptiveness to this innovation. Physicians who felt uncomfortable with EHR technology and were comfortable in delegating work adapted well to the Physician Partner model. However, they needed to learn how to effectively communicate with the Physician Partner by summarizing key elements of the history, assessment, and plans so that these components could be easily documented. In contrast, physicians who felt extremely facile with the EHR system or did not feel comfortable delegating responsibilities did not perceive benefit from the program. Some physicians found that their workflow was disrupted when they had to clarify documentation or computerized patient order entry during the visit. In our pilot, we did not assess the quality of clinical documentation or accuracy of orders entered.

We also recognized that Physician Partners require training to fulfill their responsibilities. Accordingly, we developed a curriculum that includes modules on medical note writing, medical acronyms and abbreviations, physical exam findings, and anatomy review. The curriculum also includes extensive EHR training, in which the Physician Partners complete simulations in a test environment. Despite this formal training, however, it takes time for the Physician Partner to adapt to the workflow and writing style of the individual physicians.

Retaining Physician Partners may be an issue. For some, this may be a long-term position, but for perhaps the majority, it may be a short stop along the way to another career in health care. None of the first three Physician Partners are still working in this capacity; all have pursued further health care education.

There are also costs associated with hiring additional personnel, which may be offset by increased physician productivity. These costs and offsets vary by health system, physician provider specialty, and geography. Economic models for estimating the benefit might consider downstream revenues as well, such as income from administering reimbursable medications or an increased volume of procedures performed as a result of higher patient volume. It must be recognized, however, that innovations such as Physician Partners and scribes are temporary solutions that may be obviated by better configuration of the EHR, for example, through improved voice recognition systems. Finally, Physician Partners and scribes are just one of many collaborative care and documentation innovations being implemented in primary care.^2^

Despite these limitations, the use of scribes has increased dramatically with the proliferation of organizations that train and certify medical scribes through dedicated programs.^4^ For the moment, Physician Partners and scribes may be one of the better solutions to an imperfect EHR. However, Physician Partners are not the solution for all providers and health systems. Growth will be limited to physicians who embrace this change in clinical workflow and to health care systems that are willing to invest in implementing this innovation to counteract the unintended consequences of mandating the implementation of EHRs without adequately considering the downstream effects on physician users.^5^
